# A novel selection model based on multivariate methods and arbitrary genetic parameters: a case study on tomato families

**DOI:** 10.1186/s13007-023-00992-5

**Published:** 2023-03-13

**Authors:** Peyman Eynizadeh, Hamid Dehghani, Ali Dehghani

**Affiliations:** 1 Plant Genetics and Breeding Department, Faculty of Agriculture, Tarbiat Modares University, 14115-336, Tehran, Iran; 2 Department of Plant Science, University of California, One Shields Avenue, Davis, CA 95616 USA; 3 College of Biological Sciences, University of California, One Shields Avenue, Davis, CA 95616 USA

**Keywords:** Modified analytical hierarchy process, Multipurpose selection, Repeatable selection, Single plant selection, Stability index, Stability of broad sense heritability

## Abstract

**Background:**

Selection is one of the essential skills whereby breeders reduce the population size and increase the chance of success. Various selection methods with special applications have been developed. Superior genotypes are assessed according to interesting traits, including univariate, multivariate, phenotypic, genotypic, etc.

**Methods:**

Mathematical calculation of the traits' importance based on the genetic makeup of investigated population (average degree of dominance/additive involved in the action of genes) and arbitrary genetic parameters is functional. In this paper, a general model for multivariate selection has been presented whereby the selection can be made for (a) more than one interesting trait, (b) the trait(s) with complex inheritance, (c) finding superior genotypes from among a large-scale population, (d) finding superior genotypes in segregating generations and (f) finding tolerant genotypes to stresses. This model is developed based on biometric concepts in four steps. MATLAB script is provided for the model, and users can easily apply that to identify the most suitable genotypes after data collection according to the breeding purposes.

**Results:**

The main features of this model are simplicity, precision, repeatability, and speed (improving several traits simultaneously). All the steps and the analysis of the results are explained step by step in a case study.

**Supplementary Information:**

The online version contains supplementary material available at 10.1186/s13007-023-00992-5.

## Background

Assessing genetic diversity can help the breeders to obtain important information from the population and to determine the breeding strategy. “Selection” is one of the most important skills in plant breeding which is affected by genetic diversity. In other words, maximizing the response to selection depends on optimizing the use of available genetic diversity [1]. Breeding purposes determine the characteristics of superior genotypes. Fruit yield, fruit quality, tolerance to biotic/abiotic stress, and early ripening are the most important traits that breeding programs follow. Unfortunately, it has been found in many investigations that desirable quality-related traits have a negative or insignificant correlation with performance. Also, many economic traits are polygenic with a complex inheritance pattern [2–4]. According to the symmetrical or asymmetrical distribution of genes with positive or negative effects on traits, the superior parents do not always produce superior progenies [5]. One of the ways to improve interesting traits with complex inheritance is to consider them as the dependent variable and improve them along with some other correlated traits as independent variables. The more the amounts of heritability and correlation with the dependent variable(s), the more the value of the independent variable in selection [6].

One of the reliable parameters to evaluate the variables' effect on each other is to calculate genetic correlation by the statistical designs [7]. This correlation is better than Pearson’s correlation due to the elimination of environmental effects in estimating the relationship. Broad sense heritability is a type of heritability calculated from the ratio of genetic variance to phenotypic variance and can be estimated by statistical designs [8, 9].

The stability of independent variables is an important parameter for evaluation, especially in stress conditions. The lower the effect of stress on a variable, the more stable the variable. The stability of traits and/or genotypes in different environments has been investigated by different researchers [10, 11]. Determining the stability of the traits/genotypes in different conditions by regression analysis has already been introduced and performed by other researchers [12–14].

In recent years some scientists used mathematical concepts such as artificial neural network, genetic algorithms, and the Modified Analytical Hierarchy Process (MAHP) to make the best decision [6, 15, 16]. One of the effective multivariate selection methods in plant breeding is index selection, which can be done by mathematical methods. Despite all the advantages, it is not a popular method for breeders. One of the reasons is the variability of the traits’ coefficient from one generation/condition to another [17, 18]. Therefore, considering the traits’ stability to calculate the index can minimize the changes in the traits’ coefficient. In this regard, MAHP has been developed by [6] as a practical use of AHP [19] in index selection.

In this paper, a multivariate selection model is presented whereby the traits' importance is calculated mathematically based on the arbitrary genetic parameters, the special purposes of the breeding program, and the particular competitive genotypes. The basis of the model is the characterization of the traits and genotypes before the selection by available parameters. Two new genetic parameters were introduced and used in this paper: the Stability Index (SI) and the Stability Index of Broad-Sense Heritability (SIBH). To better understand, a case study on some tomato families is presented, and the implementation of the model is explained step by step.

## Materials and methods

### Methodology

The proposed model has been developed according to the following well-known biometric concepts;

**Concept one:** It is usually important to improve yield and quality in plant breeding [5].

**Concept two:** Traits related to yield and quality are almost polygenic [5].

**Concept three:** Differences in the expression of the different genes (dominant and additive effects) caused complicated inheritance patterns of polygenic traits [4, 20].

**Concept four:** Because simple univariate selection has low repeatability and the results cannot be predicted in the next generations, multivariate selection is recommended to improve traits with complicated inheritance patterns [6, 21].

**Concept five:** In multivariate selection, traits with higher correlation with interesting traits are more important [6].

**Concept six:** Among the traits correlated with interesting traits, one with significant physiological effects is more important in selection [6].

This model is done by following steps;

**Step 1)** Determining the inheritance pattern of the interesting traits for breeding, such as fruit yield, quality, ripening time, stress tolerance, etc., by preliminary experiments or literature review.

**Step 2)** Determining all morphological, phenological and physiological traits related to interesting traits (step 1) and determining their inheritance pattern by preliminary experiments or literature review.

**Step 3)** Creating competitive conditions for genotypes and measuring all possible traits (determined in steps 1 and 2) and calculating genetic parameters effective in the selection, such as correlation with interesting traits, heritability, expected genetic advance, and stability in different conditions. Other statistical methods may be needed to calculate the parameters, such as analysis of variance, different types of correlation, distance/similarity matrix, etc.

**Step 4)** Weighting all the measured traits and scoring the competing genotypes by MAHP.

A case study on tomato families introduces the following model.

### Measured traits and abbreviations

In this experiment, 18 physiological, phenological, and morphological traits were measured according to the following descriptions;

Number of days to 50% flowering (DTF), number of days to 50% fruit formation (DFF), number of days to harvesting (DTH), plant height (H), cluster number per plant (CN), fruit number per plant (FN), fruit yield per plant (Yld), average of single fruit weight (SFW), average of single fruit volume (SFV), fruit density (FD), fruit water content (FWC), relative chlorophyll content (SPAD), fruit juice acidity (pH), fruit juice electrical conductivity (EC), total dissolved solids (TDS), fruit juice salinity (Sal), total soluble solids (TSS), and relative water content (RWC).

Plant material, experimental design, and method for measuring traits and genetic parameters are presented in detail (see Additional file 1).

### Estimations and analysis

Genetic parameters calculation was explained in detail (see Additional file 1).

The Stability Index of Broad-Sense Heritability (SIBH) and the Stability Index (SI) were introduced and used as stability parameters to determine the stability of traits in different conditions.

The SIBH is calculated by Eq. 1.

1 $$SIBH = BH_{2} /BH_{1}$$

where BH_2_ and BH_1_ are the estimated $h_{b}^{2}$ of a trait in stress and normal experiments, respectively.

The large amount of SIBH means;

- Trait changes in different conditions appear more in genetic diversity.
- Genetic diversity plays an important role in trait control, especially in stress conditions.
- Selection can be effective in improving traits.
- Thus, the large value of SIBH means a higher trait value in the selection.

Research on proteome variations has revealed that the impact of the genotype on the proteome variations may be much higher than the stress effect [22]. In such cases, the existence of a method to measure the effect of genetic diversity on the occurrence of traits like SIBH can be important.

The SI is the absolute of the linear regression coefficient of data in the normal condition (as the independent variable) and data in the stress condition (as the dependent variable). The less the amount of SI, the more the stability of the trait. This parameter is just like the simple linear regression of Eberhart and Russell (1966), with the difference that it is calculated for traits.

**Fig. 1:**
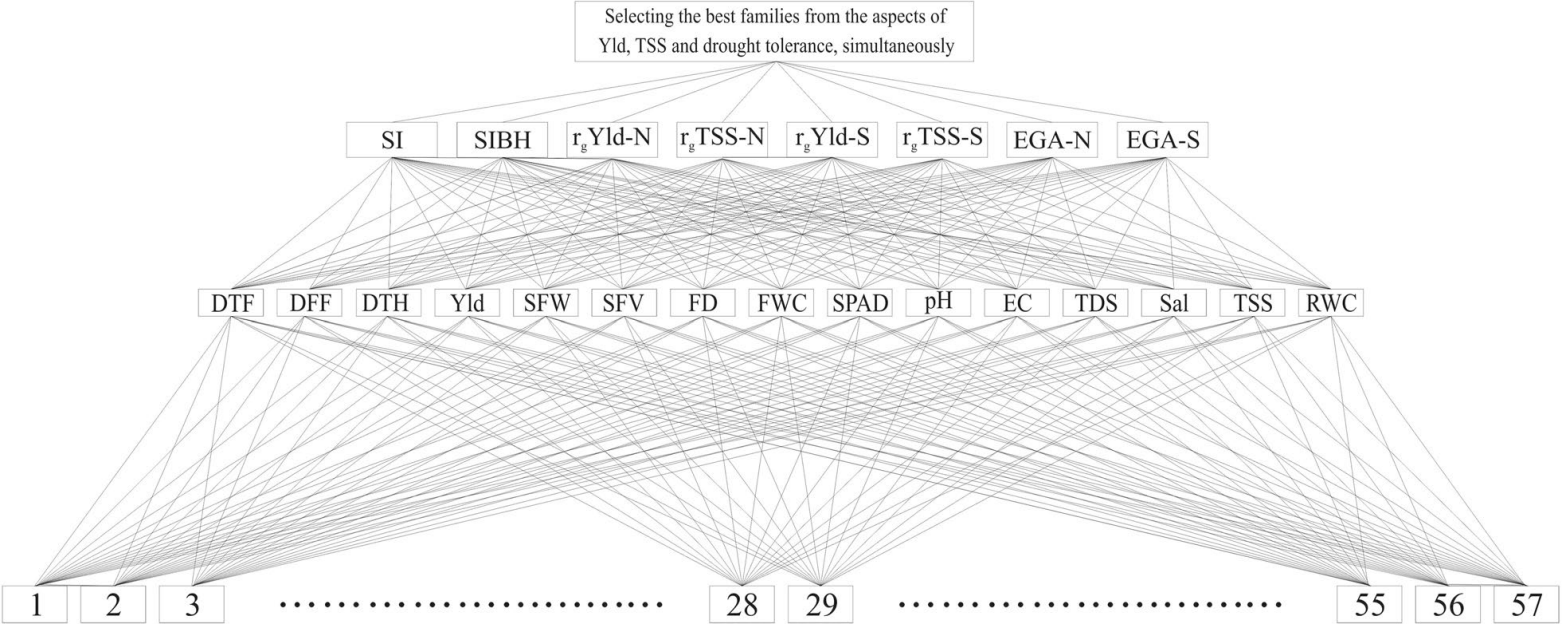
Aligning families, traits and parameters in MAHP. 1–57 integers; families DTF; Number of days to 50% flowering, *DFF* Number of days to 50% fruit formation, *DTH* Number of days to harvesting, *Yld* fruit yield per plan, *SFW* average of single fruit weight, *SFV* average of single fruit volume, *FD* fruit density, *FWC* fruit water content, *SPAD* relative chlorophyll content, *pH* fruit juice acidity, *EC* fruit juice electrical conductivity, *TDS* total dissolved solids, Sal fruit juice salinity, *TSS* total soluble solids, *RWC* relative water content. *SI* stability index, *SIBH* stability index of broad sense heritability, *r*_*g*_*Yld-N* genetic correlation with Yld in normal experiment, *r*_*g*_*TSS-N* genetic correlation with TSS in normal experiment, *r*_*g*_*Yld-S* genetic correlation with Yld in Stress experiment, *r*_*g*_*TSS-S* genetic correlation with TSS in stress experiment, *EGA-N* expected genetic advance in normal condition, *EGA-S* expected genetic advance in stress condition

MAHP was used according to [6] with the following steps:

**Step 1) Aligning traits and families:** According to Fig. 1.

**Step 2) Calculating weights of traits:** Forming trait comparison matrices based on each of the calculated genetic parameters and then calculating the eigenvectors of the resulting matrices. In the following, combining the main eigenvectors and obtaining the weights of the traits.

**Step 3) Calculating weights of families:** Forming family comparison matrices based on each trait and then calculating the eigenvectors of the resulting matrices. In the following, combining the main eigenvectors and obtaining the score of families.

The construction of pairwise comparison matrices is based on parameters (for weighting traits) or traits (for scoring genotypes). For example, in the case of T traits and P parameters, there are N consistency comparison matrices (T × T dimensions where, A_ij_ = 1/A_ji_.). The following points in constructing pairwise comparison matrices must be considered:

(1) The data must be standardized if parameters/traits have different units.

(2) Low amounts for some traits or parameters (in the presented case study: phenological traits and parameter of SI) are important. Before standardizing data, each must be transformed based on the maximum value (Eq. 2).

2 $$TD_{i} = MD{-}D_{i}$$

where TD_i_ is i^th^ transformed data, D_i_ is i^th^ data, and MD is the maximum value.

(3) Constructed comparison matrix is a consistency matrix where all its variations are compressed in an Eigenvector [23–25].

(4) The main Eigenvalue (λ) is calculated according to Eq. 3;

3 $$\left| {CM - \lambda I} \right| = 0$$

where CM is the comparison matrix, λ is the main Eigenvalue, and I is the N × N identity matrix. In consistency matrices, the main Eigenvalue is a single non-zero value.

(5) The main Eigenvector (x) related to the λ is calculated according to Eq. 4;

4 $$\left( {CM - \lambda I} \right)x = 0$$

where x is the main Eigenvector that is defined by Gaussian elimination.

In this paper, calculations of ANOVA, genetic parameters, SI, SIBH, and MAHP were done by MATLAB ver [26]. The total data and MATLAB script for calculating MAHP are presented in additional files (see Additional files 2 and 3).

## Results

The proposed model has been implemented step by step in the case study:

**Step 1)** Interesting traits were fruit yield and total soluble solids. Many related studies were previously done to determine their inheritance pattern, such as [27]. Many other researchers approved polygenic and complicated inheritance.

**Step 2)** Introduced traits in material and methods were investigated in the literature review. In addition, some previous studies were done by [6]. All of them are polygenic and are controlled by additive and dominance effects. Thus, they have a complicated inheritance.

**Step 3)** Statistical methods were used to calculate genetic parameters after data gathering from competing genotypes. ANOVA, estimating genetic correlation with Yld and TSS, SI, SIBH, EGA, and heatmap clustering were used for this step.

Tables 1 and 2 show ANOVA, $h_{b}^{2}$ and EGA for all the traits in normal and drought stress experiments, respectively. Families had significant differences in almost all traits. Regarding the three traits of H, CN, and FN, the families did not significantly differ in the two experiments. These traits showed an amount of around zero or negative amounts in parameters of ${\mathrm{H}}_{\mathrm{b}}^{2}$ and EGA. Thus, they can be ignored from the selection process. Other traits had positive and nonzero amounts of ${\mathrm{H}}_{\mathrm{b}}^{2}$ and EGA.

**Table 1:** ANOVA, broad sense heritability (${\mathrm{H}}_{\mathrm{b}}^{2}$) and Expected Genetic Advance (EGA) in normal experiment

S.O.V	DF	Mean of squares								
		DTF	DFF	DTH	H	CN	FN	Yld	SFW	SFV
Replication	2	207.462	124.936	12.848	27821.134^a^	19490.528^a^	1672.741^a^	215637.324	6144.584^b^	1084.634^b^
Families	56	140.670	149.298^a^	53.089^b^	6432.100	4202.610	303.535	3147800.590^b^	985.280	53080.267^b^
Error	112	116.718	101.864	5.925	7955.570	5145.226	379.873	552932.699	1214.939	206.868
${\mathrm{H}}_{\mathrm{b}}^{2}$	–	0.170	0.318	0.888	~ 0	~ 0	~ 0	0.824	~ 0	0.996
EGA%	–	3.474	5.320	5.556	~ 0	~ 0	~ 0	37.292	~ 0	123.350

S.O.V	DF	Mean of squares								
		FD	FWC	SPAD	pH	EC	TDS	Sal	TSS	RWC
Replication	2	0.085^a^	2.110	3.506	0.187	0.798	0.226	0.275	1.351	0.004
Families	56	0.513^b^	117.172^b^	208.135^b^	2.276^b^	9.750^b^	2.707^b^	3.320^b^	16.396^b^	0.045^b^
Error	112	0.023	13.219	23.396	0.252	1.078	0.304	0.368	1.826	0.003
${\mathrm{H}}_{\mathrm{b}}^{2}$	–	0.954	0.887	0.888	0.889	0.889	0.888	0.889	0.889	0.928
EGA%	–	87.949	27.805	23.163	32.911	50.153	49.494	49.538	60.426	27.237

^a^, ^b^ means significant in 5% and 1% probability level, respectively

*DTF* Number of days to 50% flowering, *DFF* Number of days to 50% fruit formation, *DTH* Number of days to harvesting, *H* plant height, *CN* cluster number per plant, *FN* fruit number per plant, *Yld* fruit yield per plan, *SFW* average of single fruit weight, *SFV* average of single fruit volume, *FD* fruit density, *FWC* fruit water content, *SPAD* relative chlorophyll content, *pH* fruit juice acidity, *EC* fruit juice electrical conductivity, *TDS* total dissolved solids, *Sal* fruit juice salinity, *TSS* total soluble solids, *RWC* relative water content

**Table 2:** ANOVA, broad sense heritability (${\mathrm{H}}_{\mathrm{b}}^{2}$) and Expected Genetic Advance (EGA) in drought stress experiment

S.O.V	DF	Mean of squares								
		DTF	DFF	DTH	H	CN	FN	Yld	SFW	SFV
Replication	2	218.690	211.480	14.368^a^	227.468	30.037	1.803	8475.547	85.938	235.887
Families	56	134.679^a^	125.251^b^	44.377^b^	2375.152	1591.293	124.455	999824.592^b^	406.842	18095.968^b^
Error	112	80.589	73.366	4.577	3293.174	1991.688	154.764	112924.005	397.958	306.065
${\mathrm{H}}_{\mathrm{b}}^{2}$	–	0.402	0.414	0.897	~ 0	~ 0	~ 0	0.887	0.022	0.983
EGA%	–	8.003	6.334	5.150	~ 0	~ 0	~ 0	50.721	0.775	147.304

S.O.V	DF	Mean of squares								
		FD	FWC	SPAD	pH	EC	TDS	Sal	TSS	RWC
Replication	2	0.017	31.146^a^	54.415^a^	0.271	0.833	0.228	0.289	1.718	0.006
Families	56	0.657^b^	90.521^b^	157.793^b^	2.969^b^	9.003^b^	2.512^b^	3.134^b^	18.659^b^	0.036^b^
Error	112	0.014	9.696	16.842	0.329	1.011	0.277	0.349	2.081	0.010
${\mathrm{H}}_{\mathrm{b}}^{2}$	–	0.978	0.893	0.893	0.889	0.888	0.890	0.889	0.888	0.720
EGA%	–	82.017	26.680	20.235	39.370	47.776	47.313	47.531	64.804	22.042

^a^, ^b^ means significant in 5% and 1% probability level, respectively

*DTF* Number of days to 50% flowering, *DFF* Number of days to 50% fruit formation, *DTH* Number of days to harvesting, *H* plant height, *CN* cluster number per plant, *FN* fruit number per plant, *Yld* fruit yield per plan, *SFW* average of single fruit weight, *SFV* average of single fruit volume, *FD* fruit density, *FWC* fruit water content, *SPAD* relative chlorophyll content, *pH* fruit juice acidity, *EC* fruit juice electrical conductivity, *TDS* total dissolved solids, Sal; fruit juice salinity, *TSS* total soluble solids, *RWC* relative water content

Table 3 shows some estimated genetic parameters of traits, including SI, SIBH and genetic correlation with Yld and TSS in normal and drought stress experiments. Except for SI, the larger amounts of the parameters for each trait, the larger effect on selection and the larger weight for the trait. The value of SI near zero means more trait stability in different conditions.

**Table 3:** Genetic parameters of measures traits

Traits	SI	SIBH	r_g_Yld-N	r_g_TSS-N	r_g_Yld-S	r_g_TSS-S
DTF	0.087	2.359	− 0.604	0.134	0.169	0.163
DFF	0.070	1.304	− 0.374	0.050	0.242	0.165
DTH	0.051	1.010	− 0.170	0.214	0.132	0.235
Yld	0.040	1.076	1.000	− 0.211	1.000	0.046
SFW	0.187	0.094	1.000	− 0.430	0.992	− 0.287
SFV	0.223	0.987	0.931	− 0.232	0.916	0.019
FD	0.173	1.025	− 0.808	0.363	− 0.922	0.062
FWC	0.050	1.006	0.253	− 0.171	− 0.114	0.045
SPAD	0.020	1.006	0.091	0.009	− 0.087	− 0.077
pH	0.074	1.000	0.140	0.019	0.108	− 0.088
EC	0.203	0.998	− 0.203	0.754	0.016	0.705
TDS	0.200	1.002	− 0.200	0.758	0.017	0.708
Sal	0.210	0.999	− 0.200	0.754	0.008	0.705
TSS	0.113	1.000	− 0.211	1.000	0.046	1.000
RWC	0.733	0.776	0.596	− 0.012	0.401	− 0.154

*DTF* Number of days to 50% flowering, *DFF* Number of days to 50% fruit formation, *DTH* Number of days to harvesting, *Yld* fruit yield per plan, *SFW* average of single fruit weight, *SFV* average of single fruit volume, *FD* fruit density, *FWC* fruit water content, *SPAD* relative chlorophyll content, *pH* fruit juice acidity, *EC* fruit juice electrical conductivity, *TDS* total dissolved solids, *Sal* fruit juice salinity, *TSS* total soluble solids, *RWC* relative water content

*SI* stability index, *SIBH* stability index of broad sense heritability, *r*_*g*_*Yld-N* genetic correlation with Yld in normal experiment, *r*_*g*_*TSS-N* genetic correlation with TSS in normal experiment, *r*_*g*_*Yld-S* genetic correlation with Yld in Stress experiment, *r*_*g*_*TSS-S* genetic correlation with TSS in stress experiment

**Step 4)** The selection of the remaining traits can be made on the average of the data in two experiments. MAHP has been used to weigh traits, and then scoring families based on them has been done. Figure 1 shows aligning families, traits, and parameters. Table 4 shows juxtaposed eigenvectors obtained from the pairwise comparison matrices of traits and the final weight of traits. According to the weights, the most effective traits in selection were determined as SFV, EC, TDS, Sal, and TSS, respectively. Table 5 shows the final score of families. The best families selected based on the proposed model were determined as 2, 3, 8, 14, 23, 25, 31, 39, 41, and 56, respectively. Based on the final score, these families were in the top 5% of 57 families.

**Table 4:** Eigenvectors obtained from the pairwise comparison matrix of traits and the final weights

Traits	SI	SIBH	r_g_Yld-N	r_g_TSS-N	r_g_Yld-S	r_g_TSS-S	EGA-N	EGA-S	**Final weight**
DTF	0.096	0.540	− 0.224	0.075	0.027	0.099	0.018	0.038	0.668
DFF	0.078	0.298	− 0.139	0.028	0.039	0.100	0.027	0.030	0.461
DTH	0.056	0.231	− 0.063	0.119	0.021	0.142	0.028	0.024	0.560
Yld	0.044	0.246	0.371	− 0.118	0.160	0.028	0.190	0.238	1.160
SFW	0.207	0.022	0.704	− 0.240	0.961	− 0.174	0.036	0.004	1.520
**SFV**	0.247	0.226	0.345	− 0.129	0.147	0.011	0.627	0.692	**2.167**
FD	0.192	0.235	− 0.300	0.202	− 0.148	0.038	0.447	0.385	1.051
FWC	0.055	0.230	0.094	− 0.095	− 0.018	0.027	0.141	0.125	0.560
SPAD	0.022	0.230	0.034	0.005	− 0.014	− 0.047	0.118	0.095	0.443
pH	0.082	0.229	0.052	0.011	0.017	− 0.053	0.167	0.185	0.690
**EC**	0.225	0.228	− 0.075	0.420	0.003	0.426	0.255	0.225	**1.707**
**TDS**	0.222	0.229	− 0.074	0.422	0.003	0.428	0.252	0.222	**1.704**
**Sal**	0.233	0.229	− 0.074	0.420	0.001	0.426	0.252	0.223	**1.710**
**TSS**	0.125	0.229	− 0.078	0.557	0.007	0.605	0.307	0.305	**2.057**
RWC	0.812	0.178	0.221	− 0.007	0.064	− 0.093	0.139	0.104	1.417

Bolded values are the most important traits in selection

*DTF* Number of days to 50% flowering, *DFF* Number of days to 50% fruit formation, *DTH* Number of days to harvesting, *Yld* fruit yield per plan, *SFW* average of single fruit weight, *SFV* average of single fruit volume, *FD* fruit density, *FWC* fruit water content, *SPAD* relative chlorophyll content, *pH* fruit juice acidity, *EC* fruit juice electrical conductivity, *TDS* total dissolved solids, *Sal* fruit juice salinity, *TSS* total soluble solids, *RWC* relative water content

*SI* stability index, *SIBH* stability index of broad sense heritability, *r*_*g*_*Yld-N* genetic correlation with Yld in normal experiment, *r*_*g*_*TSS-N* genetic correlation with TSS in normal experiment, *r*_*g*_*Yld-S* genetic correlation with Yld in Stress experiment, *r*_*g*_*TSS-S* genetic correlation with TSS in stress experiment, *EGA-N* expected genetic advance in normal condition, *EGA-S* expected genetic advance in stress condition

**Table 5:** Final score of investigated families

Family	1	**2**	**3**	4	5	6	7	**8**	9	10	11	12	13	**14**	15	16	17	18	19
Score	1.631	**3.299**	**2.698**	1.822	1.101	0.667	1.497	**2.915**	2.191	2.173	2.337	1.697	1.430	**2.717**	2.327	2.301	2.022	1.226	2.423
Family	20	21	22	**23**	24	**25**	26	27	28	29	30	**31**	32	33	34	35	36	37	38
Score	2.418	1.455	1.787	**2.662**	1.461	**2.673**	1.985	2.242	1.078	1.226	1.540	**3.156**	1.789	2.488	1.511	1.873	1.724	1.366	0.607
Family	**39**	40	**41**	42	43	44	45	46	47	48	49	50	51	52	53	54	55	**56**	57
Score	**2.762**	2.077	**2.806**	1.811	1.616	1.714	1.880	0.915	0.846	2.320	1.869	1.833	2.369	1.409	1.195	2.456	1.499	**2.960**	1.780

Bolded values are selected families

## Discussion

Genetic diversity in the population helps breeders increase the next generation’s average by selecting superior genotypes. Plant breeding and selection are not possible without genetic diversity. In the following, based on the mentioned concepts, it is explained how the proposed model can be used for selection. In the case study, the measured traits were weighted, the families were scored, and the model’s logic was explained by assessing the statistical and physiological relationships between the traits.

### Concepts one and two

Increasing product quality may be as important as increasing product yield in a breeding program, especially in the case of crops and vegetable breeding. Based on the type of yield and quality-related data obtained from counting or measuring, it is obvious the interesting traits are polygenic. Also, the polygenic nature of these traits can be seen in this case study.

### Concepts three and four

The dominant or additive effects of genes controlling a trait have special effects on inheritance and make it difficult to predict the selection response. Thus, these effects must be assessed before the selection process. Direct selection of traits controlled by the dominance effect may not show suitable EGA in the next generation, so direct selection cannot be useful for traits with low breeding values [27]. Assessing $h_{b}^{2}$, along with EGA, in the next generation can be more effective than $h_{b}^{2}$ alone. Indeed, EGA can help in decision-making instead of breeding value and estimating additive effect when it is impossible to calculate these parameters by the available data [27, 28].

The genetic diversity of the different families was investigated in terms of all measured traits on normal and drought stress conditions. The traits without significant diversity in the competitive families were ignored. Tables 1 and 2 show the results of ANOVA, $h_{b}^{2}$ and EGA of traits in normal and drought stress experiments, respectively. Yld and TSS were the main interesting traits, so the breeding program was done to improve them. Interesting traits had a high value of $h_{b}^{2}$ in both normal and drought stress conditions. TSS had high EGA in both normal and drought stress conditions. Yld had a medium value of EGA in stress conditions and a low value of EGA in normal conditions. Therefore, the univariate direct selection is not recommended for any of these traits in normal and drought stress conditions. Theoretically, some traits with no significant variability in different genotypes can be eliminated from the multivariate selection. For example, in tomato families, although H, CN and FN have an important physiological role in fruit yield, they cannot be used as a variable in multivariate selection because of the non-significant difference among the genotypes in both normal and drought stress conditions.

Selection of traits with higher values of $h_{b}^{2}$ and EGA can be more effective. Therefore, these values can be used as two parameters for weighting the traits.

### Concepts five and six

The more the correlation between a trait with interesting traits, the more it affects them. This effect may be positive or negative. According to Table 3, the genetic correlation between two interesting traits of Yld and TSS (dependent variables) and other traits (independent variables) in normal and drought stress conditions can be considered an important parameter. The most stable trait from normal to drought stress conditions was SPAD. The greatest amount of SIBH was seen for DTF. Thus, changes in normal and drought stress conditions appear more in genetic diversity, and selection can effectively improve that.

### The most important traits and the best families

According to Table 4, the most important traits for selection were SFV and TSS, respectively. They were the most effective traits on dependent variables. Many investigations have shown the importance of the mentioned traits on various plants [29–32]. Still, there is no research on weighting traits and determining their importance mathematically by considering their inheritance pattern and stability. Selection based on TSS can effectively improve that in the next generation because of the high EGA in normal and drought stress conditions and a good genetic correlation between that and some other independent variables. However, Yld was not an important trait for direct selection. Although Yld was one of the interesting traits, the selection of superior genotypes based on that cannot be effective in the next generations.

Suppose the results of calculating the genetic parameters of traits in different conditions are similar. In that case, selection principles in different conditions can be considered the same, and selection can be made based on the average genotypes in different conditions. If the behavior of the traits in different conditions is completely different, different breeding strategies must be considered in each condition. Tables 1 and 2 show similar behaviors of the traits in terms of $h_{b}^{2}$ and EGA in normal and drought stress conditions. Therefore, the score of genotypes can be calculated based on the average values in two conditions. Selected families by this model are the best choices to construct the next generation’s population. They will be more likely to produce a population with a high Yld, TSS, and more drought tolerant than other families. Because of the consideration of traits' stability parameters against drought stress and their inheritance pattern, selected families are more likely to construct a generation with a high yield/quality and drought stress tolerance than other families. Finally, it should be noted that, in case of a low heritability for target trait(s) and an unpredictable average of the next generation’s population, it is better to use correlated heritable traits as independent variables in multivariate selection. Therefore, the use of this model is not limited to advanced generations. This is an advantage of the proposed model, which ranks the genotypes according to several genetic parameters and is independent of the homozygosis percentage.

## Supplementary Information

**Additional file 1.** Plant material, experimental design, and how to measure traits and genetic parameters (.docx).

**Additional file 2.** Excel of data related to the case study (.docx).

**Additional file 3.** MATLAB script for calculating scores (.M).

## Data Availability

The dataset(s) supporting the conclusions of this article are included within the article and its additional files. All of the material is owned by the authors and/or no permissions are required.
