# Supplementary material for: A novel selection model based on multivariate methods and arbitrary genetic parameters: a case study on tomato families
Source: Plant Methods. 2023 Mar 13;19:25. doi: 10.1186/s13007-023-00992-5 (PMC10012525; doi:10.1186/s13007-023-00992-5)
Supplement: Supplementary file 1 — Additional file 1. Plant material, experimental design, and how to measure traits and genetic parameters (.docx). [file 13007_2023_992_MOESM1_ESM.docx]

**Plant material, experimental design and how to measured traits and genetic parameters;**

In a breeding program, after several cross-pollination and three generations of self-pollination (2016-2019), 57 S4 families were obtained with approximately 93.5% purity according to Hallauer et al. (2010). The families were investigated in two separate experiments of normal and drought stress conditions in Randomized Complete Block Design (RCBD) with three replications in 2020. At least seven plants (seedlings) were planted in each experimental plot with 50 cm × 120 cm density. The length of the growing season in the experimental area was 6 months and the climate was moderately arid. Soil texture was sandy loam, rotted manure was applied 10 t ha–1 before planting, and weeding was done manually. In normal experiment, drip irrigation was applied in 50% of Plant Available Water (PAW) to ~60 cm depth. So that, plants were not affected by any water related stress. In drought stress experiment, drip irrigation was done in 30% of PAW according to the soil texture analysis. Detection of the irrigation time had been done by sampling from a depth of 30 cm and soil moisture curve.

Morphological traits were measured at the end of the harvest on five compatible plants from each replication and then, average of samples' data recorded as replication data. Picking fruits characterizing were done in full ripening stage according to [2]. Physiological traits related to stress tolerance including, pH, EC, TDS, Sal, TSS and RWC [3]were measured in each replication on random picked up leaves/fruits and mixed together.

Tomato fruit juice was provided to use by a digital refractometer to measure TSS and express results in Brix (GMK-701 AC, Korea). Also, the pH value of the tomato juice was determined by a pH meter (HANNA HI-2020, USA). The salinity of the tomato fruit juice was determined with a conductivity meter (Model, CR-30, Colorado, Denver instrument). RWC was calculated according to Henson et al. (1981).

After data collection and hypothesis checking, analysis of variance based on RCBD was done for each experiment separately with general linear model. The broad sense heritability ($h_{b}^{2}$) and the Expected Genetic Advance (EGA) with a selected proportion of 1% were calculated based on the RCBD model with the help of Eqs. 1 to 4.

$\text{σ}_{\text{g}}^{2}=(\text{MSg}-\text{MSE})/r$ (1)

$\sigma_{p}^{2}=\sigma_{g}^{2}+\sigma_{e}^{2}/r$ (2)

$h_{b}^{2}=\sigma_{g}^{2}/\sigma_{p}^{2}$ (3)

$\text{EGA\%}=((\text{i}\times\text{h}_{\text{b}}^{2}\times\text{σ}_{\text{p}})/\bar{X})\times100$ (4)

Were$\sigma_{g}^{2}$ ,$\sigma_{p}^{2}$ and $\sigma_{e}^{2}$ are estimates of genotypic, phenotypic and error variances, respectively. MSg and MSE are genotypic and error mean square, respectively. $\bar{X}$ is the mean of the total trait.$\text{σ}_{\text{p}}$ is estimation of the phenotypic standard deviation, r is the number of repetitions and i = 1.8 is the selection intensity for the selected proportion of 1%.

Genotypic correlation between traits was calculated according to Holland (2006).

**References:**

1. Hallauer AR, Carena MJ, Miranda Filho JB de. Quantitative genetics in maize breeding. 3rd ed. New York: Springer; 2010.

2. Moneruzzaman KM, Hossain A, Sani W, Saifuddin M, Alenazi M. Effect of harvesting and storage conditions on the post harvest quality of tomato (Lycopersicon esculentum Mill) cv. Roma VF. Aust J Crop Sci. Southern Cross Publisher; 2009;3:113–21.

3. Eynizadeh P, Dehghani H. Selecting elite tomato genotypes through a modified analytical hierarchy process. Crop Pasture Sci. CSIRO; 2020;71:822–30.

4. Henson IE, Mahalakshmi V, Bidinger FR, Alagarswamy G. Genotypic Variation in Pearl Millet ( Pennisetum americanum (L.) Leeke), in the Ability to Accumulate Abscisic Acid in Response to Water Stress. J Exp Bot [Internet]. JSTOR; 1981;32:899–910. Available from: https://academic.oup.com/jxb/article-lookup/doi/10.1093/jxb/32.5.899

5. Holland JB. Estimating genotypic correlations and their standard errors using multivariate restricted maximum likelihood estimation with SAS Proc MIXED. Crop Sci. Crop Science Society of America; 2006;46:642–54.
